# The Assessment of the Paediatric Athlete

**DOI:** 10.1007/s12265-020-10005-8

**Published:** 2020-05-04

**Authors:** Guido E. Pieles, Renate Oberhoffer

**Affiliations:** 1 Congenital Heart Unit, Bristol Heart Institute, Upper Maudlin Street, Bristol, BS2 8BJ UK; 2 Department of Sports and Health Sciences, Technical University Munich, Georg Brauchle Ring, 80992 Munich, Germany

**Keywords:** Paediatric sports cardiology, Paediatric athlete, Athletic adaptation, Paediatric arrhythmia, Paediatric cardiomyopathy, Paediatric exercise

## Abstract

The success of systematic early age talent development has led to the professionalisation of youth sports academies used by clubs and governing bodies alike, and sports physicians are nowadays commonly confronted with paediatric cardiological problems. Medical cardiac care of the paediatric athlete is however in its infancy, and the international guidelines that are present for adult athletes, are not yet available. Similarly, reference data for ECG and echocardiography are incomplete. The aim of this article is to provide and introduction to the cardiac care of the paediatric athlete to facilitate healthy and above all, safe talent development, but also provide guidance on how to distinguish adaptive, beneficial cardiovascular remodelling from underlying pathology of congenital or inherited cardiovascular disease. Differences in presentation, diagnosis and treatment between childhood and adult athletes are highlighted and can educate the reader in the emerging field of paediatric sports cardiology.

## Introduction

### General Activity Recommendations for Children

Healthy children are more active than adults, and exercise and sports are a central experience of childhood and are essential for a normal physical and mental development [1]. The importance of integration of sports and exercise into the daily lives of children is reflected in international public health activity recommendations, adapted from Pieles [1] and updated [2, 3]:

### World Health Organization (WHO 2019, 5–17 Years)

- At least 60 min of moderate to vigorous physical activity daily, > 60 min provide additional health benefits. Most physical activity should be aerobic.
- Vigorous-intensity activities at least 3 days/week
- Activities that strengthen muscle and bone.

### Canadian Society for Exercise Physiology (CSEP 2012, 5–17 Years)

- 60 min of daily moderate to vigorous physical activity.
- 3 days a week of vigorous-intensity activities
- 3 days a week of activities that strengthen the musculoskeletal system
- 2 h per day maximum of sedentary screen time

### US Department of Health and Human Services (HHS, 2008, Update 2018, Children and Adolescents)

- Minimum of 60 min of moderate to vigorous physical activity daily
- Vigorous-intensity activities at least 3 days/week
- Activities that strengthen muscle and bone at least 3 days/week.

### UK Department of Health (DoH, 2011, 5–18 Years)

- Minimum of 60 min of moderate to vigorous physical activity daily
- Activities that strengthen the musculoskeletal system at least 3 days/week.
- Minimise sedentary time.

### Australian Government Department of Health, 2005, 5–18 Years)

- Minimum of 60 min of moderate to vigorous physical activity daily
- 2 h per day maximum time using electronic media for entertainment.

These recommendations aim to counteract the increasingly sedentary nature of childhood and translate the overwhelming evidence of health benefit from regular physical exercise. On the one hand, we see an alarming trend towards a sedentary lifestyle that starts in childhood. In the UK, only 14% of 13 to 15 year old boys and 9% of girls achieved recommended physical activity levels—a figure which has fallen from 28% in boys and 14% in girls in 2008. [4].

The advent of professional youth athlete academies has on the other side produced a subgroup of children who compete at an increasingly high and professional level. These paediatric athletes we define here as being between 12 and 16 years old using criteria from Araujo and Scharhag [5]. In this age group, training intensity and volume can reach that of professional adult athletes in many sporting disciplines and emerging data show that excessive training can have a detrimental effect on the developing musculoskeletal system leading to overuse injuries also in children [6]. Similarly, the syndrome of overtraining and burnout is now well recognised in elite paediatric athletes [7], and this emphasises the need for specialised medical care for the paediatric athlete population.

### Epidemiology of Disease and Sudden Cardiac Death

Data from the general population show that 2000 children die each year from sudden cardiac death (SCD) in the USA, the incidence from available studies ranges from 0.6 to 8/100,000 [8, 9]. Within the young athlete population, the prevalence of conditions associated with SCD is 0.2 to 0.7% [10]. The most recent study in a subpopulation of adolescent screened footballers determines the incidence of sudden cardiac death as high as 6.8 per 100,000 athletes screened [11]. While the overall aetiology of SCD in adolescent athletes is in general comparable to those in young adult athletes, slightly different aetiological frequencies can be explained by a different age of presentation for inherited cardiac conditions [12, 13]. Importantly, the first disease presentation in approximately 50% is sudden cardiac arrest, providing a strong argument of early detection of disease by screening [14, 15]. Despite controversy regarding the most suitable methodology to perform cardiac screening to prevent SCD [16], there is evidence that screening strategies have reduced the number of SCD in childhood, adolescent and young adult athletes [17–19]. Causes of SCD in the paediatric athlete population (Finocchiaro et al. 2016, Harmon et al. 2015, Ecart 2011) are in 44% some form of structural heart disease such as congenital cardiac anomalies or cardiomyopathies, nonstructural heart disease accounts for the remaining 56%.

## Cardiac Adaptation to Training in the Paediatric Athlete

Knowledge on physiological changes to athletic training in childhood are still limited, but recent metaanalysis data show that adaptation occurs in athletes as young as 12 years [20]. A challenge remains to differentiate training related cardiac changes from somatic growth and pubertal development with little research available for guidance. Training effects in adolescent athletes are well established [21], but physiological gender–related maturation and development particular during puberty with growth, hormone dependent bone and muscle mass maturation, changes of motoric entities (endurance, speed, strength, balance), age and weight related heart rate, blood pressure and cardiac output, respiratory capacity and peak oxygen uptake all need to be taken into account when differentiating training progress from growth and maturation. Structural and functional cardiac adaptations to exercise are slightly less pronounced in prepubertal athletes compared with adult athletes and are however present [20], and the cardiovascular response to regular training includes decrease of resting heart rate, increase of cardiac output, ventricular cavity size and wall thicknesses. There is the suggestion that HR-mediated cardiac output increase to endurance exercise is dominant mechanism in peripubertal children [22]. Most pronounced cardiovascular adaptations appear in classical endurance sports like rowing, triathlon and swimming [23].

## Diagnostic Tools and Their Specific Use in the Paediatric Athlete

There has been much of discussion on when, to which extent and if at, all the young athlete, especially before puberty, should be screened before participating in competitive sports, and the current data is insufficient to provide evidence-based guidance. However, even if the tragic event of sudden cardiac death is rare, many inherited diseases can be suspected, defined or excluded by experienced paediatric cardiologists. Recommendations for cardiac screening in adolescent athletes are modelled on adult guidelines and do not specifically target the adolescent athlete population also drawing on data obtained in studies on adult athletes [24]; this also includes the Association for European Paediatric and Congenital Cardiology (AEPC) working group sports cardiology, physical activity and prevention document [25].

### Medical, Family History and Physical Examination

Medical and family history remain the mainstay also in the assessment of the paediatric athlete and should include number and extent of weekly training and the beginning of the career as an athlete in order to interpret ECG findings, echocardiography and exercise test. Family history assessment of cardiovascular disease of either congenital, inherited or acquired cardiac disease and risk factors (dyslipidaemia, hypertension, diabetes) as well as sudden cardiac death are central. Symptoms of palpitations, exercise-dependent respiratory symptoms, dizziness and syncope should be evaluated in detail. Recent infections can have a low but appreciable risk myocarditis and need to be accurately assessed to give advice on training intensity and volume. Based on physician experience, pyrexia, should mandate a break, whereas in simple upper airway only infection light endurance training can be continued [26, 27]. Nutritional assessment is of importance for the paediatric sports cardiologist as athlete anorexia is no uncommon especially in gymnastics with a female predominance [28], additionally nutritional supplements can be arrhythmogenic by adrenergic overstimulation, in particular found with the use of energy drinks [29].

Besides a standard cardiac examination, it is important to exclude congenital lesions detectable with specific heart murmurs or pulse and BP differences in hand and feet (e.g. coarctation). Important is also the assessment for syndromes; many are associated with cardiac disease (e.g. signs of connective tissue disease, Marfan syndrome, Williams syndrome, Turner syndrome). This is particularly important when assessing paralympic athletes.

### 12-Lead ECG

A resting ECG should be part of preparticipation screening also in paediatric athletes. Interpretation follows routine age and height dependent reference values [30] with respect to heart rate, time intervals, heart axis, negative precordial T waves and signs of hypertrophy. The current ECG interpretation guidelines for adults [31] show acceptable accuracy in the paediatric athletes [32] and should be used here, but it has to be taken into consideration that valid only from 14 years of age. Thus, in younger age, there is a grey zone regarding young athlete’s ECG in particular when assessing T wave inversions in anterior but also inferior and lateral leads and in different ethnicities. While chronological age of the athlete helps to differentiate between a physiological juvenile and pathological T wave morphology, biological age is a more reliable denominator [33].

### Echocardiography

Echocardiography is not a primary screening tool but remains the primary diagnostic imaging modality also in paediatric cardiology. Studies to assess morphological cardiac adaptations in adolescent athletes and provide reference values have been published that confirm effects of athletic training for the left [34] and at a smaller scale for the right ventricle [35]. Data correlating echocardiographic data to training volume and intensity, ethnicity, gender and age is still missing for the paediatric age group. As in the interpretation of the ECG, echocardiographic examinations should be in the hands of experienced paediatric cardiologists in particular as a first time assessment needs to exclude congenital heart disease and coronary artery lesions [36]. General paediatric echocardiographic guidelines should be followed [37], which also include data on reference values for common functional echocardiographic parameters [38] and morphology [39], quantitative values should be indexed for BSA and *z* scored. Advanced functional modalities where reference data for healthy children are available such as speckle tracking echocardiography [40] or exercise stress echocardiography [41, 42] should be considered in specific cases.

### Exercise Assessment

#### Cardiopulmonary Exercise Testing (CPET)

Cardiopulmonary exercise testing (CPET) is an important secondary assessment tool in paediatrics not only to objectively assess overall functional exercise capacity but also to investigate for specific metabolic, respiratory or cardiac pathology [43, 44] such as pulmonary vascular disease, dysfunction of the autonomic nervous system and peripheral defects in oxygen transport and oxygen utilization at the working muscle. This makes it an important tool for risk stratification in children with congenital or acquired heart disease [45] but also to exclude exercise pathology in paediatric athletes. CPET should always be combined with exercise 12-lead ECG testing to allow assessment of underlying exercise-related arrhythmias. It is paramount that supervising personnel should be trained and familiar with the paediatric population to encourage maximal effort and deliver reliable test results [46, 47]. Specific considerations are that children do not always attain a true VO_2_max, [48] and submaximal measures such as ventilatory anaerobic threshold (VAT) [49] or oxygen uptake efficiency slope (OUES) [50] can be equally good markers for endurance performance. CPET can also indirectly pick up reduced stroke volume caused by ventricular dysfunction in cardiomyopathies or congenital heart disease.

#### Functional Cardiac Imaging During Exercise Stress

Exercise stress echocardiography has recently emerged as a functional assessment tool to unmask subclinical pathology. Indications include assessment of systolic and diastolic function, valve function, myocardial exercise reserve, subtle wall motion abnormalities or dyssynchrony. Quantification of function during exercise should include less load dependent parameters such myocardial deformation (strain) and tissue Doppler imaging as they can provide information on force-frequency relationship and myocardial contractility reserve during exercise stress and paediatric reference data exist [41, 42]. Exercise stress is superior to dobutamine stress in the assessment of the athlete as it assesses ventricular function in relation to other physiological exercise adaptations and can be combined with cardiopulmonary exercise testing. Exercise stress cardiac magnetic resonance (CMR) is indicated when assessing cardiac volumes, function and wall motion abnormalities during exercise in athletes [51].

### Mobile Monitoring

Mobile monitoring is becoming a diagnostic tool of choice accounting for the athlete specific and chosen training environment, portability and prolonged monitoring during exercise with potentially higher pick up rates of pathology (e.g. arrhythmias). Cableless mobile and app-based devices are available for clinical use in children and early data on feasibility and diagnostic value are encouraging [52]. Mobile monitoring in the paediatric athlete should be used to investigate cardiac symptoms during exercise such as paroxysmal symptoms of palpitations, dizziness and syncope, postintervention (e.g. arrhythmia ablation) monitoring and risk stratification in pathologies (e.g. cardiomyopathies, congenital heart disease).

### Cardiac Magnetic Resonance and Computed Tomography

As in the adults, CMR allows window independent imaging, accurate delineation of detailed anatomy including 3-D reconstruction, tissue characterization and is the gold standard in ventricular and regurgitant volume quantification in children. Adult athlete recommendations on when to perform cross-sectional imaging to investigate potential pathology in athletes should be followed [53]. Size- and gender-specific paediatric centiles are available for both echocardiographic measurements and cardiac MRI [54] [55, 56]. Cardiac CT is the imaging modality of choice in the delineation of small anatomical structures such as coronary arteries and collateral arteries and for imaging parenchymal lung pathology. The main disadvantage of CT is lower temporal resolution and radiation, which calls for judicious use of CT in the paediatric athletes although modern technology has reduced the exposure to radiation.

## Specific Diagnostic Considerations in the Paediatric Athlete

Hereditary and acquired arrhythmia disorders and cardiomyopathies are the major cause for sudden cardiac death also in the paediatric athlete. Adult competitive sports participation guidelines should be adhered to in the diagnosis and management of arrhythmias [57] as well as cardiomyopathies [58]. It is however important to remember that an underlying inherited cardiac pathology previously dormant may present in particular during puberty when the organism is exposed to the rapid growth and hormonal changes that accompany this stage in life. In particular for cardiomyopathies, the main differential diagnoses of athlete’s heart also in paediatric athletes, age and mode of presentation can be different from that in adults. The developing nature of cardiomyopathies requires serial annual evaluation as a single point assessment does not exclude disease. Index case and family cascade genotyping in paediatric cardiomyopathies should be performed when clinically indicated. However, age of independent—and legal—capacity to consent to genetic testing is debated, and a close collaboration with a geneticist and genetic councillor is recommended. No specific paediatric guidelines for paediatric cardiomyopathy genetic testing exist, but there is emerging evidence, that genotyping can help in risk stratification (reviewed in [59]). In all paediatric athletes with a suspected cardiomyopathy, serial annual evaluation is paramount and should include ECG, echocardiogram, exercise testing and CMR.

### Hypertrophic Cardiomyopathy

Diagnosis of HCM in the paediatric athlete population remains a challenge as the majority of cases remain phenotypically dormant in childhood. Family history is central and first line 12-lead ECG screening should follow the published international ECG criteria for athletes. Family history, ECG, Echocardiography and CMR are imaging tools of choice. LV hypertrophy should be categorised as abnormal if the LV end-diastolic wall thickness *z* score > 2. End-diastolic LV diameter is reduced in the majority of phenotypic paediatric HCM [60]; however, more common are mild and developing or subclinical phenotypes in childhood. As in adults, abnormal echocardiographic diastolic tissue Doppler parameters or myocardial systolic strain can be first indicators of an evolving phenotype [60]. Data on the use of detraining in the paediatric athlete population to ascertain a diagnosis of HCM is still absent.

### Arrhythmogenic Right Ventricular Cardiomyopathy

Clinical diagnosis of arrhythmogenic right ventricular cardiomyopathy (ARVC) in the paediatric population is challenging, and the disease can be concealed in childhood and often mimics changes seen in the healthy young athletic population [61]. Diagnostic criteria have evolved solely based on evidence from the affected adult patient population [62] and are less sensitive and specific in paediatric ARVC [63]. Echocardiographic revised task force criteria in particular rarely trigger suspicion in children and adolescents as they rely on adult RV diameters and are less sensitive and specific in paediatric ARVC [64], and enlarged cardiac diameters in paediatric athletes can mimic cardiomyopathic changes [35]. Recent data suggest that additional modalities such as 2-D strain are more sensitive in assessing adolescents with ARVC [63]. CMR is the diagnostic imaging modality of choice also in children, but CMR findings in adult ARVC such as fatty infiltration and fibrosis are of limited value in children and focus lies on ventricular function, regional wall motion abnormalities and *z* scores of RV and LV dimensions. Arrhythmic activity is highly variable in adolescent patients with ARVC [65], serial arrhythmia monitoring and exercise testing is important. Little data on the onset and incidence of LV involvement in children and adolescents exist. Importantly, as in adults, exercise can unmask or exacerbate disease and paediatric athletes with phenotypic ARVC should be restricted following adult recommendations [58].

### Left-Ventricular Noncompaction Cardiomyopathy

Left-ventricular noncompaction cardiomyopathy (LVNC) in childhood shows an undulating and heterogeneous phenotype of different severity [66]. LV hypertrabeculation is not uncommon in the healthy adult athlete [67], no comparable studies exist for the childhood athlete and a differentiation between physiological trabeculation and LVNC disease in this age group remains a challenge.

Suspicion of LVNC on echocardiography in the paediatric athlete warrants careful further assessment by CMR and rhythm monitoring. Mild hypertrabeculation in the setting of normal function, without CMR features such as wall motion abnormalities or fibrosis, and without evidence of rhythm abnormalities can be regarded as a normal phenomenon, but serial monitoring is advised. Advanced echocardiographic imaging tools such as speckle tracking strain imaging can help detect disease [68].

### Care of the Paediatric Athlete in the Future

The rapid development and professionalisation of youth sports academies demand increased diligence to safeguard the development of the paediatric athlete. The significant progress achieved in adult sports cardiology can guide assessment of the paediatric athlete, but adult cardiac preparticipation screening and management recommendations cannot be unequivocally applied to the paediatric heart. Somatic growth, psychocognitive maturation with particular communicational and also ethical and legal considerations require an interdisciplinary approach involving paediatricians, paediatric cardiologists and sports medicine professionals as well as coaching staff with the young athlete in the centre.

Future research should focus on the development of gender, growth and age-specific normative assessment criteria for ECG and echocardiogram to increase diagnostic accuracy. Most importantly, development of training pathways and a stronger engagement of paediatric and sports governing bodies are required, as too few paediatricians and paediatric cardiologists are sufficiently trained to provide expert opinion on the paediatric athlete [69]. Consequently, an approach requiring synergy between paediatric and sports cardiologists, exercise physiologists, policy makers and sports organisations is required to develop paediatric cardiac monitoring tools and protocols, eventually working towards a child athlete centered specialty (paediatric sports cardiology) that matches the sports professionalism of the current and future paediatric athlete.
